# Induction of tumours by administration of N-dibutylnitrosamine and derivatives to infant mice.

**DOI:** 10.1038/bjc.1977.95

**Published:** 1977-05

**Authors:** K. Fujii, S. Odashima, M. Okada

## Abstract

Pulse doses of N-dibutylnitrosamine(DBN), N-butyl-N-(4-hydroxybutyl)nitrosamine(BBN) and N-butyl-N-(3carboxypropyl)nitrosamine(BCPN) suspended in 1% gelatin, were administered s.c. to infant CDF1 mice, and the experiment terminated at one year of age. Tumours were induced in lungs and liver. The incidences of lung adenomas were 73-95% in all treated mice, with no sex differences. Hepatocellular adenomas and a carcinoma were found with an incidence of 81% (21/26) in DBN, 59% (13/22) in BBN, and 32% (9/28) in BCPN-treated males and the incidence was 23% (5/22) in DBN-treated females. Only one papilloma of the fore-stomach was induced in mice treated with DBN. These results indicated that the s.c. administration of DBN, BBN, and BCPN induced tumours of the lung and liver, but no tumours of the urinary bladder, under these experimental conditions. The carcinogenic effect on mice at the treated dose level was DBN greater than BBN greater than BCPN.


*Br. J. Cancer* (1977) **35**, 610

### INDUCTION OF TUMOURS BY ADMINISTRATION OF N-DIBUTYLNITROSAMINE AND DERIVATIVES TO INFANT MICE

K. FUJII*, S. ODASHIMA† AND M. OKADA‡

*From the *Department of Pathology, Institute of Basic Medical Science, University of Tsukuba, Sakura, Niihari-Gun, Ibaraki, Japan 300–31, †Department of Chemical Pathology, National Institute of Hygienic Sciences, Tokyo, ‡Tokyo Biochemical Research Institute, Tokyo*

Received 27 September 1976 Accepted 1 December 1976

**Summary.—Pulse doses of N-dibutylnitrosamine(DBN), N-butyl-N-(4-hydroxybutyl)nitrosamine(BBN) and N-butyl-N-(3-carboxypropyl)nitrosamine(BCPN) suspended in 1% gelatin, were administered s.c. to infant $CDF_1$ mice, and the experiment terminated at one year of age. Tumours were induced in lungs and liver. The incidences of lung adenomas were 73–95% in all treated mice, with no sex differences. Hepatocellular adenomas and a carcinoma were found with an incidence of 81% (21/26) in DBN, 59% (13/22) in BBN, and 32% (9/28) in BCPN-treated males and the incidence was 23% (5/22) in DBN-treated females. Only one papilloma of the fore-stomach was induced in mice treated with DBN. These results indicated that the s.c. administration of DBN, BBN, and BCPN induced tumours of the lung and liver, but no tumours of the urinary bladder, under these experimental conditions. The carcinogenic effect on mice at the treated dose level was DBN > BBN > BCPN.**

N-DIBUTYLNITROSAMINE(DBN), N-butyl - (4 - hydroxybutyl)nitrosamine(BBN), and N-butyl-N-(3-carboxypropyl)nitrosamine(BCPN) are potent carcinogens among the dialkylnitrosamines, in their ability to induce tumour of urinary bladder in rodents, when the administration is started in young animals (Druckrey *et al.*, 1964; Ivankovic and Bucheler, 1968; Ito *et al.*, 1969; Bertram and Craig, 1970, 1972; Wood, Flaks and Clayson, 1970; Hashimoto, Suzuki and Okada, 1972, 1974; Okada and Hashimoto, 1974). Druckrey *et al.* (1964) have reported a selective incidence of urinary bladder tumours in BD rats administered with DBN and its hydroxylated derivative, BBN. In mice, Wood *et al.* (1970) have reported that 3 injections of 1 $\mu$l DBN to infant animals induced tumours of liver and lung rather than of urinary bladder. Okada and Suzuki (1972) have studied the metabolism of BBN in rats, and found that a majority of BBN administered orally to rats was excreted in the urine as BCPN, which selectively induced urinary bladder tumour in rats (Hashimoto *et al.*, 1972). Recently, the interesting finding was reported by Hashimoto and Kitagawa (1974) that rat epithelial cells of urinary bladder were transformed by BCPN or BBN with urea in tissue culture.

In this paper, the carcinogenic activity of DBN and two of its derivatives, BBN and BCPN, have been studied in newborn mice, and comparison was based on the affected tissues. To date, no comparable report on incidences of tumours in DBN-, BBN-, and BCPN-treated mice, when carcinogen was administered at birth, is available.

This report is a part of "Neoplastic response of newborn mice to chemicals".

### MATERIALS AND METHODS

*Animals.*—Pregnant female BALB/c mice mated with DBA/2 males were supplied by Dr K. Suzuki, Institute of Medical Science, University of Tokyo, Japan. After birth, (BALB/c $\times$ DBA/2) $F_1$ mice ($CDF_1$) were nursed by their mother, and after weaning they were maintained on CE-2 pellet diet (CLEA Japan Inc., Tokyo) and water *ad libitum.*

*Chemicals.*—N-Dibutylnitrosamine(DBN), N - butyl - N - (4 - hydroxybutyl) - nitrosam - ine(BBN), and N-butyl-N-(3-carboxypropyl)-nitrosamine(BCPN) were synthesized by Dr M. Okada, Tokyo Biochemical Research Institute, and the purity of these chemicals was analysed using thin-layer chromatography, infra-red nuclear magnetic resonance and mass spectra. Gelatin was purchased from Difco Laboratories, Detroit, Mich. Each test chemical was prepared freshly in 1% gelatin solution by using a magnetic stirrer at room temperature.

*Animal treatment.*—Newborn $CDF_1$ mice within 24 h after birth were injected s.c. on the back with 0·03 ml of 1% gelatin solution containing 158 $\mu$g DBN (105 $\mu$g/g body wt), 87 $\mu$g BBN (58 $\mu$g/g body wt), or 188 $\mu$g BCPN (125 $\mu$g/g body wt), respectively, and thereafter once a week for 3 weeks (a total of 4 injections). The doses used were the maximum tolerated dose (MTD) of each chemical in newborn $CDF_1$ mice, and the MTD was determined by the method described elsewhere (Fujii *et al.*, 1976).

Animals were weaned at one month of age and separated by sex. Five or fewer animals were housed in each plastic box with sawdust bedding in an environmentally controlled room. They were observed daily for mortality and sickness, and all animals were weighed monthly. The study was terminated at one year in all groups, and surviving animals necropsied. Complete necropsies were performed on moribund and dead mice, or on those killed at the termination of the experiment. Tissues were fixed in 10% neutralized formalin solution, sectioned at 3 $\mu$m, and stained routinely with haematoxylin and eosin. Special stains were used on restricted occasions.

*Statistical analysis.*—Statistical analysis of tumour incidence was made with the Chi-square test.

### RESULTS

### *Weaning rates and survival rates*

Experimental groups, number of surviving animals and total tumour incidence are listed in Table I.

A total of 200 newborn mice from 29 dams were separated randomly into 4 groups, including a vehicle control. Of these, 93% (142/153) of the animals in the Groups 1–3 were weaned at one month of age, compared with 77% (36/47) in the control. Eighty-seven per cent (126/142) of animals survived at the termination of the study at one year, and 97% (35/36) in the control.

### *Incidence of tumours*

Before termination of the experiment, 17 animals died or were killed when moribund. Most of them died of bronchopneumonia, or urinary tract inflammation. Of these, 4 animals bore lung adenomas and/or hepatocellular adenomas. The first appearance of a tumour was at 39 weeks.

The overall incidences of tumours in Groups 1–3 were statistically significant (77–95%; $\chi^2 = 19{\cdot}43$–$40{\cdot}30$, $P < 0{\cdot}005$) compared with the control (0 ♀ and 17% ♂, respectively). Though the overall incidences in each group were similar, the effect on Group 3 (BCPN) appeared less, and the difference in incidence between the groups was not statistically significant ($\chi^2 = 3{\cdot}08$, $P > 0{\cdot}05$). The overall incidences were confined to the lung and liver.

### *Lung tumour*

Incidences of lung tumour in Groups 1–3 were 73 to 95% as shown in Table II. They were not linked to sex, although the incidences in the females were slightly greater than in the males.

Most of the tumour nodules in Groups 1–3 were multiple, and the average number of tumours per animal was higher in Group 2 (BBN). All lesions were diagnosed as lung adenomas. The multiplicity of tumours was higher in Groups 2 and 3 than in Group 1, as shown in Table II.

TABLE I.—*Survival and Tumour Incidence in $CDF_1$ Mice Given N-Dibutylnitrosamine, N-Butyl-(4-hydroxybutyl)nitrosamine, or N-Butyl-(3-carboxypropyl)nitrosamine by s.c. Injections to Newborn*

| Group | Chemical | Dose injected | No. of animals injected | No. of litters | No. of animals weaned | Sex | No. of survivors at risk (wks) 4 | 10 | 20 | 30 | 40 | 50 | Tumour-bearing animals No. | (%)* |
|---|---|---|---|---|---|---|---|---|---|---|---|---|---|---|
| 1 | DBN | 158 μg × 4 | 52 | 7 | 48 | M | 26 | 23 | 23 | 22 | 22 | 22 | 22 | (85) |
| | | | | | | F | 22 | 22 | 22 | 22 | 22 | 22 | 20 | (91) |
| 2 | BBN | 87 μg × 4 | 46 | 7 | 44 | M | 22 | 22 | 22 | 21 | 20 | 19 | 19 | (86) |
| | | | | | | F | 22 | 22 | 22 | 22 | 21 | 20 | 21 | (95) |
| 3 | BCPN | 188 μg × 4 | 55 | 8 | 50 | M | 28 | 23 | 23 | 23 | 22 | 22 | 24 | (86) |
| | | | | | | F | 22 | 21 | 21 | 21 | 21 | 21 | 17 | (77) |
| 4 | Vehicle control | 0·03 ml | 47 | 7 | 36 | M | 18 | 18 | 18 | 18 | 17 | 17 | 3 | (17) |
| | | | | | | F | 18 | 18 | 18 | 18 | 18 | 18 | 0 | |

Animals were killed 52 weeks after the start of the experiment.
* Percentages based on the number of animals weaned at 4 weeks.

TABLE II.—*Organ Distribution of Tumours*

| Group | Chemical | Sex | Lung tumours No. | % | No./animal | Liver tumours No. | % | No./animal | Other tumours No. | % | Animals with lung *and* liver tumours |
|---|---|---|---|---|---|---|---|---|---|---|---|
| 1 | DBN | M | 19 | 73 | 1·6 | 21 | 81 | 10·2 | 0 | — | 18 |
| | | F | 19 | 86 | 2·5 | 5 | 23 | 2·5 | 1* | 5 | 5 |
| 2 | BBN | M | 19 | 86 | 3·1 | 13 | 59 | 2·8 | 0 | — | 13 |
| | | F | 21 | 95 | 3·3 | 0 | — | — | 0 | — | 0 |
| 3 | BCPN | M | 22 | 79 | 2·5 | 9 | 32 | 0·4 | 0 | — | 9 |
| | | F | 17 | 77 | 2·5 | 0 | — | — | 0 | — | 0 |
| 4 | Vehicle control | M | 2 | 11 | 0·1 | 1 | 6 | 0·1 | 0 | — | 0 |
| | | F | 0 | — | — | 0 | — | — | 0 | — | 0 |

Percentages based on number of animals at weaning.
* Papilloma of the fore-stomach.

*Liver tumour*

Liver tumours were predominantly in the males (32–81%), but there were a few tumours in the females (23%) of Group 1. The incidences were statistically significant ($\chi^2 = 4{\cdot}55$–$24{\cdot}09$, $P < 0{\cdot}05$). Vehicle control showed 6% (1/18) liver tumours in males and none in females. The incidence and average number of liver tumours per animal were higher in Group 1 (DBN) males than in Groups 2 and 3 males (BBN, BCPN) as shown in Table II. The incidences and the average number of tumour nodules per animal were in the order of DBN > BBN > BCPN, regardless of the molar dose of chemical and the solubility of chemical in animal tissues.

All except one tumour were classified as hepatocellular adenomas. The exception, in the males of Group 1 (DBN), was a hepatocellular carcinoma occupying the entire left lateral lobe, and the lesion measured 18 mm by 13 mm. Microscopically, the tumour cells were arranged in a trabecular pattern and the cell arrangements were irregular, with no metastatic foci in any remote organs.

*Other tumours*

There was only one papilloma of the fore-stomach, in a male of Group 1 (DBN).

*Other lesions than tumours*

A few pathological lesions found in mice in this experiment were bronchopneumonia, hepatitis, urinary tract inflammation and otitis media.

The urinary tracts were carefully examined and, in a few cases, thickened mucosa of the urinary bladder without round-cell infiltration was found, but there was no other specific finding for either carcinogen-treated or control groups.

### DISCUSSION

Our results indicate that s.c. injection of DBN, and its two derivatives BBN and BCPN, induced tumours of the lung and liver of mice, but not urinary bladder, when the injection was given on Day 1 of life and followed by 3 weekly injections. In one study, newborn mice treated with DBN developed tumours of the liver and lung (Wood *et al.*, 1970), but in this study DBN, BBN and BCPN also induced such tumours. There was no sex difference in the incidence of lung tumour, but liver tumours were mainly in male mice.

In the report by Wood *et al.* (1970), 1 $\mu$l of DBN in newborn (IF $\times$ C57)$F_1$ mice was 900 $\mu$g (if the sp. grav. of DBN was 0·9). However, a total dose 632 $\mu$g of DBN (158 $\mu$g $\times$ 4) in our experiment was 4·27 times lower than that (900 $\mu$g $\times$ 3) by Wood *et al.*, regardless of the strain differences.

One aim of this experiment was to develop an appreciable yield of urinary bladder carcinomas. However, in the present effort, we failed to induce urinary bladder tumour in mice. Available reports deal with a high and selective incidence of urinary bladder tumour when the administration of DBN and its metabolites was given s.c. or orally to adult rats and mice (Druckrey *et al.*, 1964; Ivankovic and Bucheler, 1968; Ito *et al.*, 1969; Bertram and Craig, 1970, 1972; Wood *et al.*, 1970; Hashimoto *et al.*, 1972, 1974). However, no bladder tumours have yet been reported to be induced in newborn mice with a limited amount of chemical carcinogen. We have confirmed this with DBN and its derivatives BBN and BCPN.

Recently, one study showed that mucosal cells of the urinary bladder in rat were made neoplastic with the combination of urea and either BCPN or BBN *in vitro* (Hashimoto and Kitagawa, 1974). This result suggests that urea in the bladder may also play an important role in transforming mucosal cells of urinary bladder into neoplastic cells *in vivo*. The relative concentrations of urea and carcinogen may be a factor in affecting mucosal cells.

The target tissues in newborn mice differ from those in adults (Takayama and Imaizumi, 1969). It may be reasonable to presume that the differences in target tissues of adult and infant mice can be due to strain, age, variety of chemical susceptibility in cells, duration of chemical exposure, absence or bypassing of the activation steps of chemicals in newborn, slow excretion of the chemical, and ineffective amounts of carcinogen in urinary bladder due to longer retention of chemical in the body.

This study emphasizes that age difference at administration of carcinogen to mice, especially newborn, can vary their responses to carcinogenic chemicals.

The authors wish to thank Dr Y. Hashimoto (Tohoku University) and Dr R. S. Yamamoto (National Cancer Institute, NIH, USA) for reading the manuscript.

This project was supported in part by Grants in Aid for Cancer Research from the Ministry of Education, Science and Culture of the Japanese Government.

### REFERENCES

Bertram, J. S. & Craig, A. W. (1970) Induction of Bladder Tumours in Mice with Dibutylnitrosamine. *Br. J. Cancer*, **24**, 352.

Bertram, J. S. & Craig, A. W. (1972) Specific Induction of Bladder Cancer in Mice by Butyl-(4-hydroxybutyl)-nitrosamine and the Effects of Hormonal Modifications on the Sex Difference in Response. *Eur. J. Cancer*, **8**, 587.

Druckrey, H., Preussmann, R., Ivankovic, S., Schmidt, C. H., Mennel, H. D. & Stahl, K. W. (1964) Selektive Erzeugung von Blasenkrebs an Ratten durch Dibutyl- und N-Butyl-N-butanol(4)-nitrosamin. *Z. Krebsforsch.*, **66**, 280.

Fujii, K., Kurata, H., Odashima, S. & Hatsuda, Y. (1976) Tumor Induction by a Single Subcutaneous Injection of Sterigmatocystin in Newborn Mice. *Cancer Res.*, **36**, 1615.

Hashimoto, Y., Suzuki, E. & Okada, M. (1972) Induction of Urinary Bladder Tumors in ACI/N

Rats by Butyl(3-carboxypropyl)nitrosamine, a Major Urinary Metabolite of Butyl-(4-hydroxy-butyl) nitrosamine. *Gann*, **63**, 637.

HASHIMOTO, Y., SUZUKI, K. & OKADA, M. (1974) Induction of Urinary Bladder Tumors by Intra-vesicular Instillation of Butyl(4-hydroxybutyl) nitrosamine and Its Principal Urinary Metabolite, Butyl(3 - carboxypropyl) nitrosamine in Rats. *Gann*, **65**, 69.

HASHIMOTO, Y. & KITAGAWA, H. S. (1974) *In vitro* Neoplastic Transformation of Epithelial Cells of Rat Urinary Bladder by Nitrosamines. *Nature, Lond.*, **252**, 497.

ITO, N., HIASA, Y., TAMAI, A., OKAJIMA, E. & KITAMURA, H. (1969) Histogenesis of Urinary Bladder Tumors Induced by N-Butyl-N-(4-hydroxybutyl)nitrosamine in Rats. *Gann*, **60**, 401.

IVANKOVIC, S. & BUCHELER, J. (1968) Leber- und Blasen Carcinome beim Meerschweinchen nach Di-n-butylnitrosamin. *Z. Krebsforsch.*, **71**, 183.

OKADA, M. & HASHIMOTO, Y. (1974) Carcinogenic Effect of N-Nitrosamines Related to Butyl(4-hydroxybutyl)nitrosamine in ACI/N Rats, with Special Reference to Induction of Urinary Bladder Tumor. *Gann*, **65**, 13.

OKADA, M. & SUZUKI, E. (1972) Metabolism of Butyl(4-hydroxybutyl)nitrosamine in Rats. *Gann*, **63**, 391.

TAKAYAMA, S. & IMAIZUMI, T. (1969) Carcinogenic Action of N-Nitrosodibutylamine in Mice. *Gann*, **60**, 353.

WOOD, M., FLAKS, A. & CLAYSON, D. B. (1970) The Carcinogenic Activity of Dibutylnitrosamine in IF × C57 Mice. *Eur. J. Cancer*, **6**, 433.


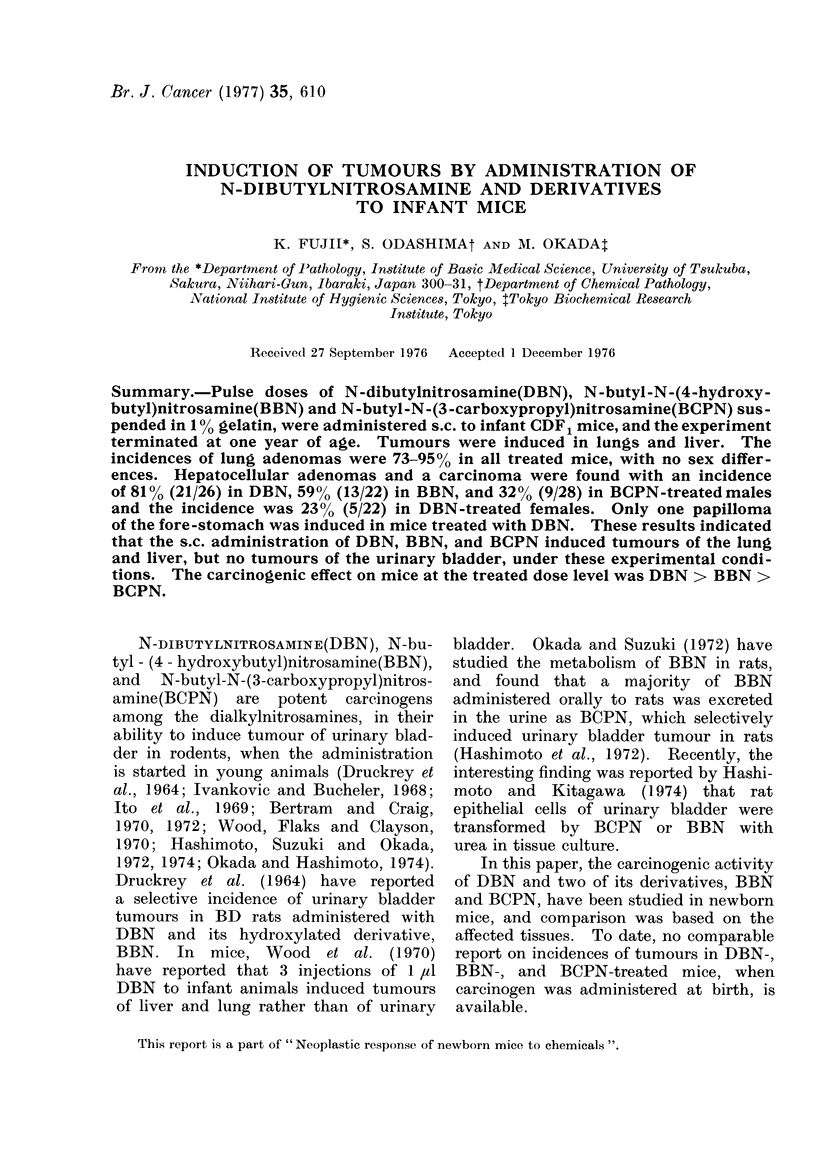

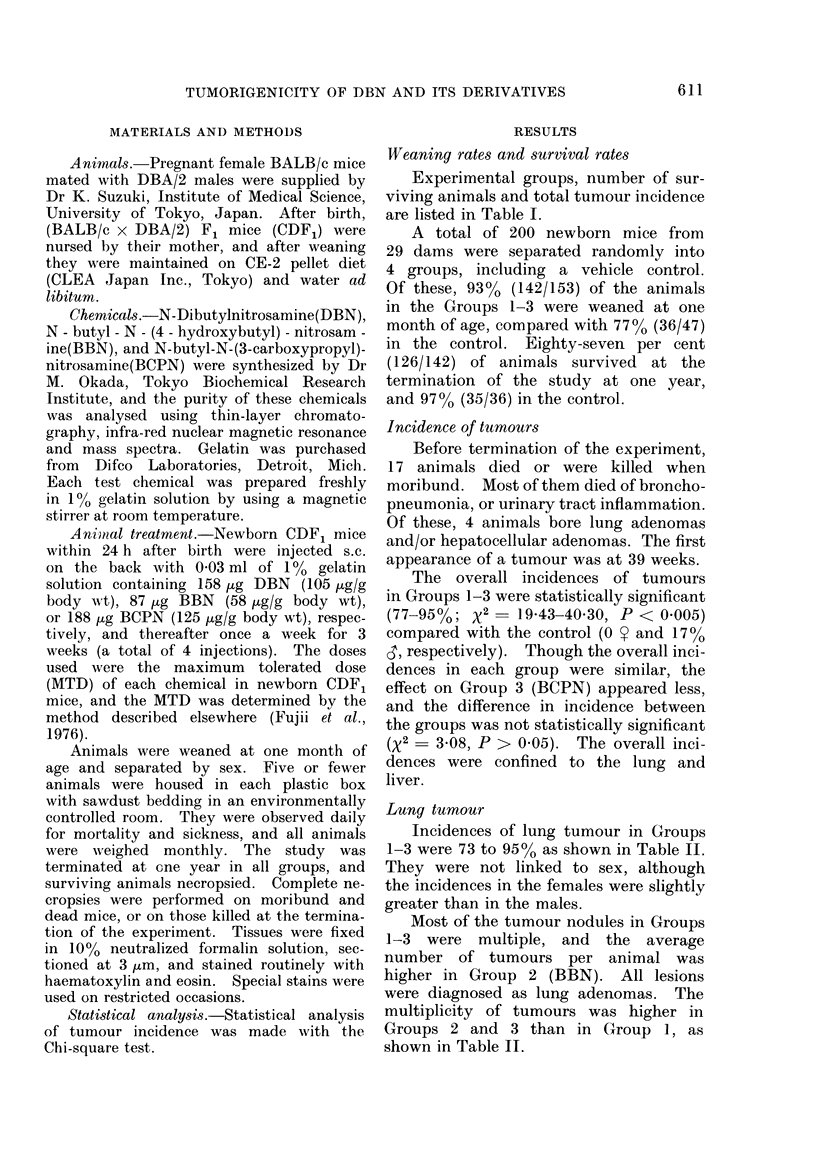

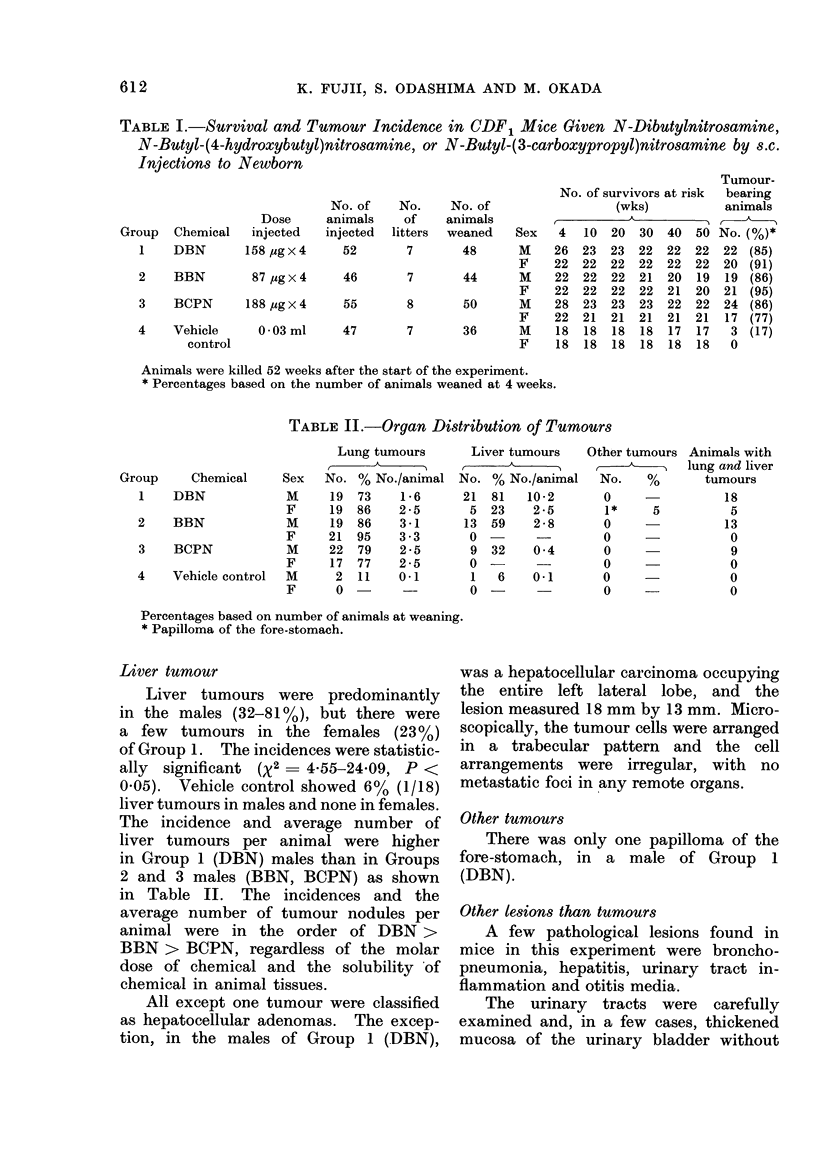

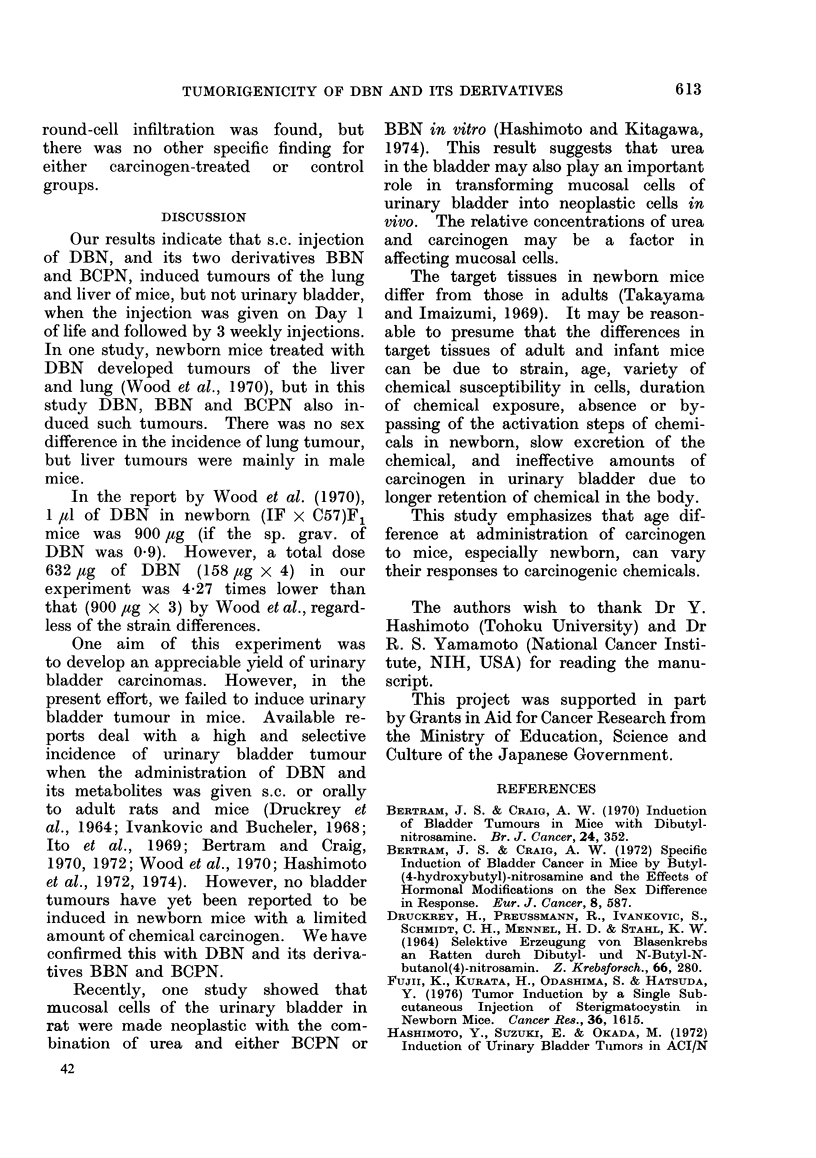

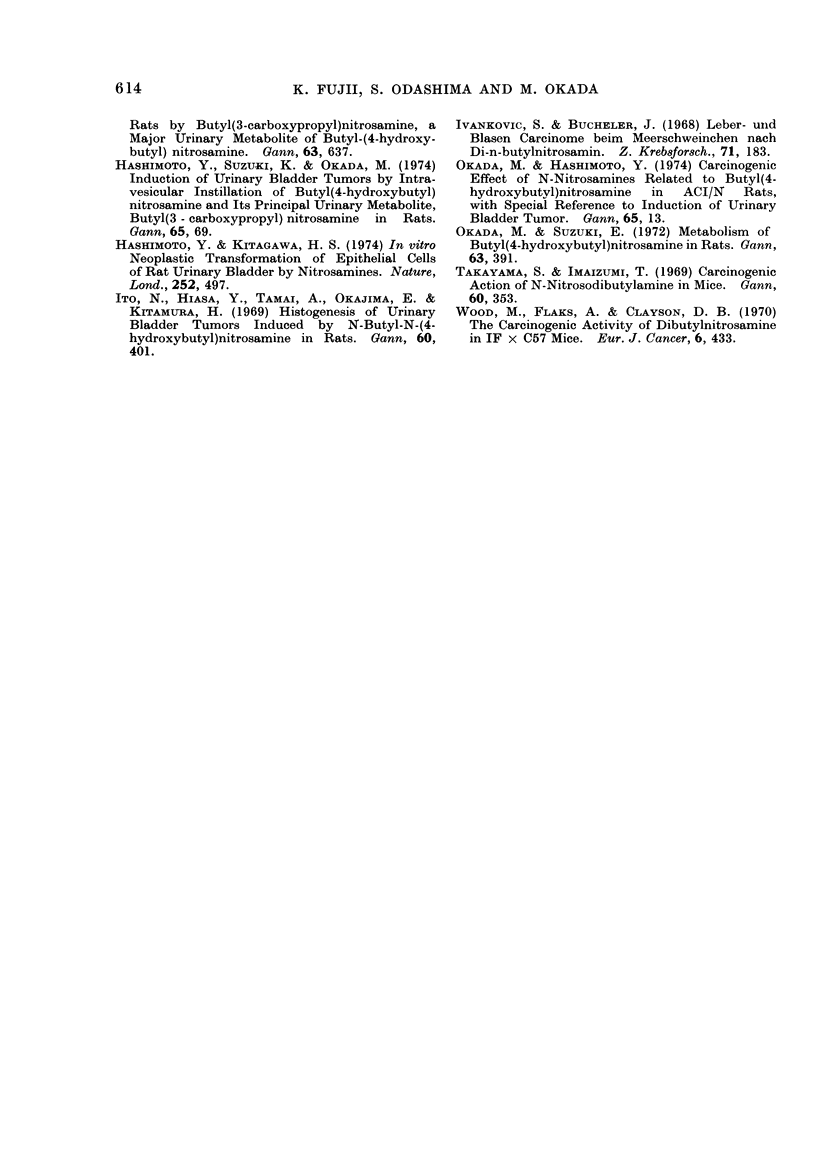


## References

[OCR_00503] Bertram J. S., Craig A. W. (1970). Induction of bladder tumours in mice with dibutylnitrosamine.. Br J Cancer.

[OCR_00508] Bertram J. S., Craig A. W. (1972). Specific induction of bladder cancer in mice by butyl-(4-hydroxybutyl)-nitrosamine and the effects of hormonal modifications on the sex difference in response.. Eur J Cancer.

[OCR_00515] DRUCKREY H., PREUSSMANN R., IVANKOVIC S., SCHMIDT C. H., MENNEL H. D., STAHL K. W. (1964). SELEKTIVE ERZEUGUNG VON BLASENKREBS AN RATTEN DURCH DIBUTYL- UND N-BUTYL-N-BUTANOL(4)-NITROSAMIN.. Z Krebsforsch.

[OCR_00522] Fujii K., Kurata H., Odashima S., Hatsuda Y. (1976). Tumor induction by a single subcutaneous injection of sterigmatocystin in newborn mice.. Cancer Res.

[OCR_00546] Hashimoto Y., Kitagawa H. S. (1974). In vitro neoplastic transformation of epithelial cells of rat urinary bladder by nitrosamines.. Nature.

[OCR_00528] Hashimoto Y., Suzuki E., Okada M. (1972). Induction of urinary bladder tumors in ACI-N rats by butyl(3-carboxypropyl)nitrosoamine, a major urinary metabolite of butyl-(4-hydroxybutyl)nitrosoamine.. Gan.

[OCR_00538] Hashimoto Y., Suzuki K., Okada M. (1974). Induction of urinary bladder tumors by intravesicular instillation of butyl(4-hydroxybutyl)nitrosoamine and its principal urinary metabolite, butyl(3-carboxypropyl)nitrosoamine in rats.. Gan.

[OCR_00552] Ito N., Hiasa Y., Tamai A., Okajima E., Kitamura H. (1969). Histogenesis of urinary bladder tumors induced by N-butyl-N-(4-hydroxybutyl)nitrosamine in rats.. Gan.

[OCR_00559] Ivankovic S., Bücheler J. (1968). Leber- und Blasen-Carcinome beim Meerschweinchen nach Di-n-butylnitrosamin.. Z Krebsforsch.

[OCR_00564] Okada M., Hashimoto Y. (1974). Carcinogenic effect of N-nitrosoamines related to butyl(4-hydroxybutyl)nitrosoamine in ACI-N rats, with special reference to induction of urinary bladder tumors.. Gan.

[OCR_00571] Okada M., Suzuki E. (1972). Metabolism of butyl(4-hydroxybutyl)nitrosoamine in rats.. Gan.

[OCR_00576] Takayama S., Imaizumi T. (1969). Carcinogenic action of N-nitrosodibutylamine in mice.. Gan.

[OCR_00581] Wood M., Flaks A., Clayson D. B. (1970). The carcinogenic activity of dibutylnitrosamine in IF x C57 mice.. Eur J Cancer.

